# Overexpression of *acdS* in *Petunia hybrida* Improved Flower Longevity and Cadmium-Stress Tolerance by Reducing Ethylene Production in Floral and Vegetative Tissues

**DOI:** 10.3390/cells11203197

**Published:** 2022-10-11

**Authors:** Aung Htay Naing, Jova Riza Campol, Mi Young Chung, Chang Kil Kim

**Affiliations:** 1 Department of Horticultural Science, Kyungpook National University, Daegu 41566, Korea; 2 Department of Agricultural Education, Sunchon National University, 413 Jungangno, Suncheon, Jeonnam 540-950, Korea

**Keywords:** ACC deaminase, cadmium (Cd) stress, flower longevity, ethylene production, gene expression, plant growth, reactive oxygen species

## Abstract

The role of *acdS*, which encodes the 1-aminocyclopropane-1-carboxylic acid (ACC) deaminase enzyme, in extending flower longevity and improving tolerance to cadmium (Cd) stress was assessed using transgenic *Petunia hybrida* cv. ‘Mirage Rose’ overexpressing *acdS* and wild-type (WT) plants. The overexpression of *acdS* reduced ethylene production in floral tissue via suppression of ethylene-related genes and improved flower longevity, approximately 2 to 4 days longer than WT flowers. Under Cd stress, *acdS* significantly reduced Cd-induced ethylene production in vegetable tissues of transgenic plants through suppression of ethylene-related genes. This resulted in a lower accumulation of ethylene-induced reactive oxygen species (ROS) in the transgenic plants than in WT plants. In addition, expression of the genes involved in the activities of antioxidant and proline synthesis as well as the metal chelation process was also higher in the former than in the latter. Moreover, Cd accumulation was significantly higher in WT plants than in the transgenic plants. These results are linked to the greater tolerance of transgenic plants to Cd stress than the WT plants, which was determined based on plant growth and physiological performance. These results highlight the potential applicability of using *acdS* to extend flower longevity of ornamental bedding plants and also reveal the mechanism by which *acdS* improves Cd-stress tolerance. We suggest that *acdS* overexpression in plants can extend flower longevity and also help reduce the negative impact of Cd-induced ethylene on plant growth when the plants are unavoidably cultivated in Cd-contaminated soil.

## 1. Introduction

Petunias have been increasingly used as a bedding plant in the landscape industry due to their wide range of colors and shapes [1]. However, this plant has been reported as highly sensitive to ethylene because high ethylene production rapidly shortens its flower longevity [1,2,3] and inhibits plant growth [4]. As a bedding plant, they are grown directly on natural soil in open fields, which makes it impossible to control ethylene overproduction as done in other cut flowers using ethylene inhibitors [5]. ACC deaminase (ACCD), encoded by the *acdS* gene, breaks down the ethylene precursor ACC into ammonia and α-ketobutyrate in all higher plants [6,7]. Previous studies reported that overexpression of *acdS* in tomato and canola significantly reduced ethylene production [8,9,10,11], whereas *acdS* significantly delayed fruit ripening of tomato by reducing ethylene production [10]. Recently, we also observed that overexpression of *acdS* significantly reduced ethylene production in the leaves of the transgenic petunia cv. ‘Mirage Rose’ [4]. However, it remains unknown whether the *acdS* overexpression reduces ethylene production in floral tissues and improves flower longevity of the transgenic petunia. Therefore, it is interesting to investigate the role of *acdS* in extending of flower longevity of the transgenic petunia by measuring ethylene production and expression of its related genes in the floral tissues.

Recently, agricultural lands contaminated with heavy metals (HMs) have been continuously increasing due to increased industrialization and application of agrochemicals worldwide [12]. Among HMs, the area of soil contaminated with cadmium (Cd) may be high because the world production of Cd-containing products has increased by approximately 20,000 tons per year [13]. In addition, as Cd is a non-biodegradable heavy metal, it can persist for a long time in the soil [14]. When plants were grown in HM-contaminated soil, they turned on ethylene biosynthesis and signaling pathways to mediate metal toxicity [15,16]. However, when the toxicity was more severe, the ethylene levels in the plants were higher, and plant growth was inhibited [17]. To date, Cd-induced ethylene production and deleterious effects of the induced ethylene on plant growth have been reported in many plant species [15,16,18,19,20,21,22,23,24,25]. Ethylene induction by Cd has been associated with enhanced expression of ethylene biosynthesis genes, such as the *ACS* gene in soybean [16] and *ACS* and/or *ACO* genes in barley [15] and *Arabidopsis* [20,24,25]. Moreover, an increase in ACS enzyme activity has been observed in *Brassica juncea* and *Triticum aestivum* plants exposed to Cd [19,22]. Cd did not affect plant growth of the *Arabidopsis* when *acs2* and *acs6* were knockout [24]. Arteca and Arteca [18] observed that Cd-induced ethylene production was highest in leaves and floral organs. Due to the Cd-induced ethylene production and consequent plant growth inhibition, the growth of petunia cultivated in Cd-contaminated soil can be severely affected. Plants inoculated with plant growth-promoting bacteria (PGPB) expressing *acdS* showed reduced ethylene production and improved plant growth under Cd stress [26,27,28]. Similarly, transgenic plants overexpressing *acdS* also exhibit improved tolerance to HM stress [8,9,11,29]. As mentioned above, we recently developed transgenic petunias expressing *acdS*, and their tolerance to the abiotic stresses (cold, drought, and salt stress) was observed [4]. However, we did not investigate whether the transgenic petunia plants could tolerate Cd stress.

HM-induced reactive oxygen species (ROS) were observed in many plant species [22,30,31,32]. Liu et al. [33] suggested that ROS interact with HM-induced ethylene in plants because ROS accumulation is linked to Cd-induced ethylene production in tomato plants. In addition, the activities of antioxidants [(catalase (CAT), superoxide dismutase (SOD), and peroxidase (POD)] and proline can be altered under HM stress because plants utilize these activities to scavenge ROS induced by HM stress [12,22,30,31,32]. Moreover, Schellingen et al. [24] observed the involvement of ethylene signaling in Cd-induced ROS signaling in *Arabidopsis*. Therefore, in this study, we evaluated the flower longevity of transgenic petunia cv. ‘Mirage Rose’ overexpressing *acdS* along with WT plants by detecting ethylene production and the expression levels of ethylene biosynthesis and signaling genes. In addition, we exposed these plants to Cd and investigated their tolerance to Cd stress by evaluating plant growth and physiological performance, ethylene content, ROS accumulation, and Cd accumulation. Furthermore, the molecular mechanism underlying the role of *acdS* in Cd-stress tolerance was investigated by assessing the transcriptional regulation of genes involved in ethylene biosynthesis and signaling, antioxidant and proline metabolism, and metal chelation.

## 2. Materials and Methods

### 2.1. Plant Materials

Transgenic petunia cv. ‘Mirage rose’ overexpressing *acdS* (lines: T-1, T-5, T-10, and T-12) and their T_2_ seeds were produced in our previous work [4]. The T_2_ seeds were used as source material for the evaluation of flower longevity and tolerance to cadmium (Cd) stress.

### 2.2. Measurement of Flower Longevity and Ethylene Production

Seeds of the T_2_ transgenic and WT plants were grown in plastic pots containing peat-based soil in a greenhouse until flowering. Before assessment of their flower longevity, flowers that opened on the same day were initially marked as done by Xu et al. [1,2]. When the petals showed rolling, the flower longevity was recorded. Thirty flowers (as three replications) were measured from each transgenic line and WT. Ethylene production in the floral tissues was also measured as done by Xu et al. [1,2]. The petals (approximately 100 mg) were sampled at the three different stages (initial, open, and fully-open stages), and they were placed in a glass tube (50 mL) and sealed with a rubber septum for 24 h. Next, ethylene production in each tube was measured using gas chromatography (GC-2010; Shimadzu, Tokyo, Japan). Three different flowers were collected from each transgenic line and WT (three replicates) for the measurement of ethylene production.

### 2.3. Expression Analysis of Ethylene Biosynthesis and Signaling Genes

Total RNA was extracted from the petals of the transgenic and WT plants, which were sampled at the three different stages (initial, open, and fully-open stages). Reverse transcription was performed as previously described [4]. Transcript levels of the ethylene biosynthesis genes [ACC synthase 1 (*ACS1*) and ACC oxidase 1 (*ACO1*)] and receptor genes [ethylene resistant 2 (*ETR2*) and ethylene response sensor 2 (*ERS2*)] in the petals were analyzed relative to that of the tubulin gene (reference gene). The relative gene expression was calculated using the quantitative comparative cycle threshold method. The PCR conditions along with the primers used for the detection of the genes are listed in Supplementary Table S1. Three biological samples were used for each analysis.

### 2.4. Cd-Stress Treatment

Surface sterilization of the transgenic and WT seeds were performed as done by Naing et al. (2021a). The sterilized transgenic seeds were germinated on hormone-free Murashige and Skoog (MS) basal medium containing 3.0% sucrose, 0.7% plant agar, and 1.0 mg/L of phosphinothricin (PPT), but the WT seeds were germinated on the medium without PPT. For the transgenic seeds, PPT was added to the germination medium in order to allow the only transgenic seeds to germinate. The cultures were placed in a culture room at a temperature of 25 °C, with a photoperiod of 16 h, and light intensity of 50 µmol m^−2^ s^−1^ for 30 days. The seedlings were then stressed with Cd by culturing them in glass bottle (400 mL) containing MS liquid medium (70 mL), which comprised an initial concentration of Cd (25 µM). After 10 days of culture, the seedlings were transferred to the same liquid medium containing a higher Cd concentration (50 µM) for the next 10 days, followed by further transferring to the medium containing the highest Cd concentration (100 µM) for another 10 days, as done by Ai et al. [12]. For the control, seedlings were cultured on MS liquid medium without Cd. The culture condition was as described above. Thirty seedlings with uniform size were selected from the transgenic lines and WT for the stress experiment. The experiment was repeated three times. After the stress period, 15 seedlings, each cultured in MS liquid media with or without Cd, were used to measure plant growth traits, such as plant height, fresh weight, number of leaves, and leaf size. In addition, physiological and biochemical traits associated with Cd-stress tolerance and expression levels of the genes related to ethylene biosynthesis and signaling, antioxidant and proline activities, and metal chelation were analyzed.

### 2.5. Measurement of SPAD Values and Relative Water Content (RWC)

The leaves of the plants exposed to Cd and control conditions for 30 days were chosen for measurement of SPAD values. The values were measured using a chlorophyll meter (SPAD-502, Minolta). RWC was measured according to the protocol described by Ai et al. [12]. Each measurement contained 10 leaves, and 30 leaves were used for three replicates.

### 2.6. Analysis of Stomatal Density

The sixth leaves from the tops of the plants subjected to Cd and control conditions for 30 days were collected. Stomatal numbers in different leaves were counted based on the method described by Naing et al. [4].

### 2.7. Measurement of Ethylene Production

The sixth and seventh leaves (approximately 100 mg each) were collected from the plants exposed to Cd and control conditions for 30 days. Ethylene production in the leaves was measured as described above. The measurements were performed three times using three different samples.

### 2.8. Detection of Cd Concentration

For determination of Cd concentration in the leaves, the leaves of the plants subjected to Cd stress conditions for 30 days were collected and oven-dried. The dried samples were then ground with a pestle to obtain a fine powder. Next, the powder (~0.3 g) was digested with a mixture of acid (HNO_3_ + HClO_4_, 5:1 *v*/*v*). Following this, Cd concentration in dried leaves was detected by ICP-OES (Optima-8300 DV; PerkinElmer, Inc., Waltham, MA, USA). The detection was conducted three times, and the values are expressed in ppm.

### 2.9. Detection of Hydrogen Peroxide (H_2_O_2_) Accumulation

Accumulation of hydrogen peroxide (H_2_O_2_) in the leaves of the plants subjected to Cd for 30 days was histochemically detected, as performed by Kumar et al. [34]. Briefly, the leaves derived from the Cd-stressed plants were immersed in a tube containing 3, 3′-diaminobenzidine (DAB) staining solution (Sigma-Aldrich, Burlington, MA, USA). Next, the tubes were kept for 12 h in the dark at 25 °C. The samples were then soaked in absolute ethanol in a 65 °C water bath for 20 min in order to remove chlorophyll for clear visualization of H_2_O_2_. Three different biological samples were used for this analysis.

### 2.10. Detection of the Genes at Transcript Level Involved in Ethylene Biosynthesis and Signaling Pathways, Antioxidant and Proline Activities, and Metal Chelation

Total RNA was isolated from the leaves of the plants subjected to Cd and control conditions for 30 days. Reverse transcription was performed using 1 µg of total RNA and an oligo (dT)20 primer. The transcript levels of the genes involved in ethylene biosynthesis and signaling (*ACS1*, *ACO1*, *ETR2*, and *ERS1*), antioxidant and proline metabolism [superoxide dismutase (*SOD*), catalase (*CAT*), peroxidase (*POD*), *Osmotin*], and metal chelation [glutathione S-transferase (*GST*) and phytochelatin synthase (*PCS*)] were detected using a StepOne Plus^TM^ (Thermo Fisher Scientific, Waltham, MA, USA). The primers and PCR conditions used for the detection of genes are listed in Supplementary Table S1. Three independent biological samples were used for the detection.

### 2.11. Statistical Analysis

Data were statistically analyzed using SPSS version 11.09 (IBM Corporation, Armonk, NY, USA). The results are presented as mean ± standard error. The least significant difference tests were performed to compare the means, and the significance was set at *p* < 0.05.

## 3. Results

### 3.1. Flower Longevity of WT and Transgenic Plants

**Figure 1:**
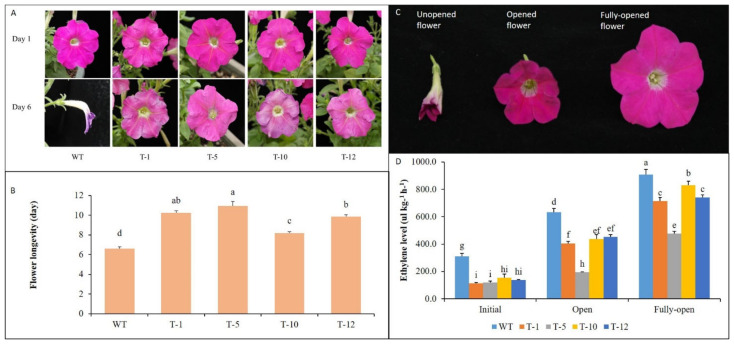
*acdS* overexpression improves flower longevity of petunia cv. ‘Mirage Rose’. Comparison of the status of flower senescence (**A**) and flower longevity (**B**) of wild-type (WT) and transgenic plants. Illustration of three different flower stages (**C**) and ethylene production in the stages (**D**) of WT and transgenic plants. Data represent the means of three replicates and error bars indicate standard error. Means with the same letters are not significantly different by least significant difference test (LSDT, *p* < 0.05).

When assessing flower longevity of WT and transgenic plants, the WT flowers exhibited early petal-rolling, specifically on 6th day after opening (DAO), but the transgenic flowers remained open for the next 2 to 4 days, depending on the transgenic lines (Figure 1A). This indicated that overexpression of *acdS* significantly improved flower longevity in petunias. However, the flower longevity was slightly or significantly varied among the transgenic lines, whereas that of T-5 line was approximately 11 days, and those of T-1, T-10, and T-12 lines were 10.24, 8.18, and 9.84 days, respectively (Figure 1B). When detecting ethylene levels in the three different stages of flowers (Figure 1C), ethylene levels detected in WT flower were significantly higher than those in the transgenic flowers for all stages (Figure 1D). The flower longevity was associated with ethylene production levels in the flowers. The ethylene levels continuously increased until the fully-opening stage in all flowers; however, those in the WT flowers were significantly higher than those in the transgenic flower at the open and fully-opening stages (Figure 1D). In addition, ethylene levels observed in T-5 for both open and fully-open stages were notably lower than those in the other transgenic lines, which was also linked to the greatest longevity of its flowers among the other transgenic flowers. Moreover, at the fully-opening stage, the ethylene level in T-10 was significantly higher than those in the T-1 and T-12 lines, and this also supported the shorter longevity of the former than the latter.

### 3.2. Expression of Ethylene Biosynthesis and Receptor Genes in Floral Tissues

**Figure 2:**
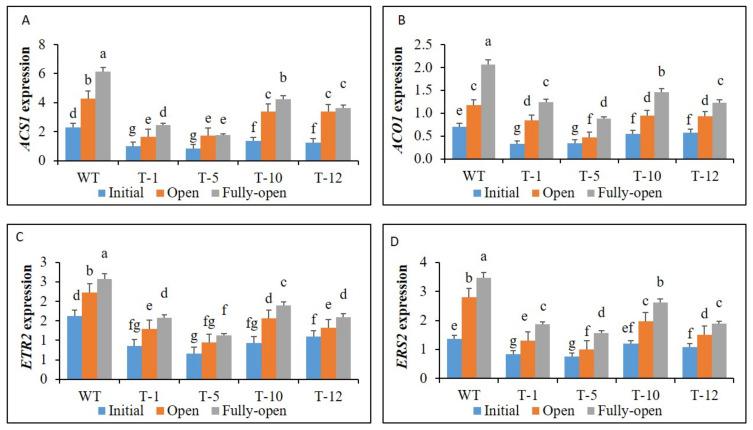
Transcript levels of ethylene biosynthesis (**A**,**B**) and signaling (**C**,**D**) genes in the three different stages of WT and transgenic petunia cv. ‘Mirage Rose’ flowers. Data represent the means of three replicates, and error bars indicate standard error. Means with the same letters are not significantly different by least significant difference test (LSDT, *p* < 0.05).

The ethylene production observed at the different flowering stages or between WT and transgenic plants or within the transgenic lines was associated with expression patterns of ethylene biosynthesis genes (*ACS1* and *ACO1*) and receptor genes (*ETR2* and *ERS2*). Because their expression levels detected in the fully-opening stage were higher than those in the opened stage, and those observed in the initial stage were the lowest (Figure 2A–D). In addition, their transcript levels expressed in WT were significantly higher than those in transgenic plants. Moreover, those expressed in T-5, which exhibited the lowest ethylene production among the transgenic lines, were also slightly or significantly lower than those in the other transgenic lines, whereas their expression levels in T-10, particularly at the fully-opened stage that produced the highest ethylene level, were the highest.

### 3.3. Plant Growth and Physiological Performance under Control and Cd Stress Conditions

**Figure 3:**
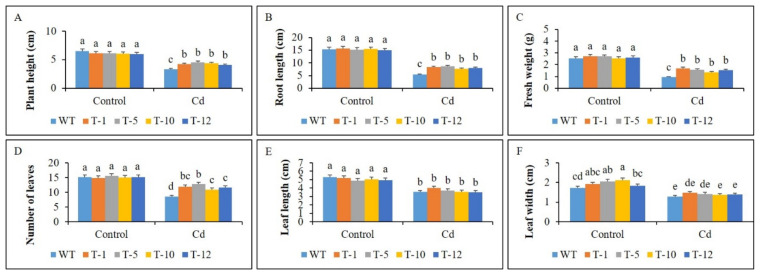
Comparison of plant growth traits (**A**–**F**) in WT and transgenic petunia cv. ‘Mirage Rose’ plants under control and cadmium (Cd) stress conditions. Data were collected on the 30th day of the experiment. Data represent the means of three replicates and error bars indicate standard error. Means with the same letters are not significantly different by least significant difference test (LSDT, *p* < 0.05).

**Figure 4:**
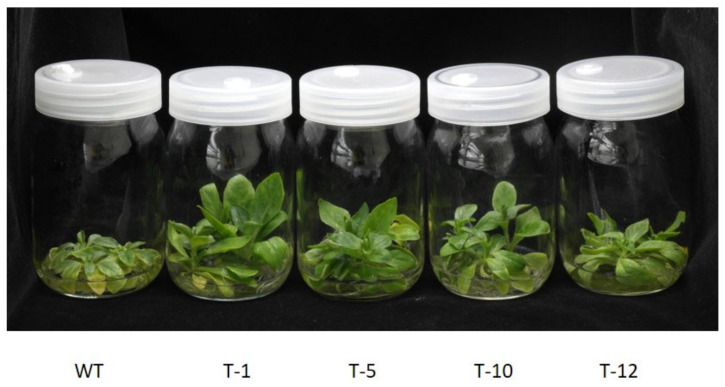
Comparison of plant growth status of WT and transgenic petunia cv. ‘Mirage Rose’ plants under cadmium (Cd) stress conditions. This image was taken on the 30th day of the experiment.

Plant growth and physiological performance of the transgenic lines and WT were evaluated after they have been subjected to Cd stress and control conditions for 30 days. Their growth performance was observed to be similar under control conditions. This was validated by their growth traits (leaf size, root length, fresh weight, and plant height) as they were not significantly different from each other. However, growth inhibition was observed when exposed to Cd stress because most of the growth traits in both WT and transgenic plants were inferior to those observed in control conditions (Figure 3A–F). The growth inhibition was severer in WT plants than in the transgenic plants, and this was linked to greater suppression of the growth traits in the former than in the latter (Figure 3A–F). These results indicated that transgenic petunia overexpressing *acdS* had a stronger tolerance to Cd stress than WT, which reflects better plant growth performance of the former than the latter under Cd stress (Figure 4).

**Figure 5:**
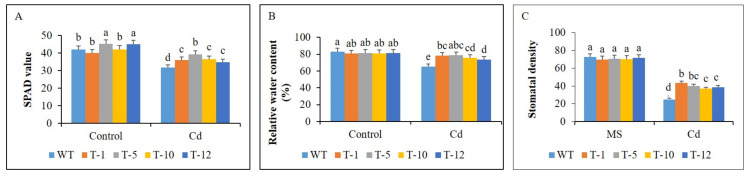
Comparison of plant physiological traits (**A**–**C**) in WT and transgenic petunia cv. ‘Mirage Rose’ plants under control and cadmium (Cd) stress condition. Data were collected on the 30th day of the experiment. Data represent the means of three replicates, and error bars indicate standard error. Means with the same letters are not significantly different by least significant difference test (LSDT, *p* < 0.05).

Plant physiological traits [such as stomatal density, RWC, and SPAD values] that are important for plant growth were assessed in the plants under both control and Cd-stress conditions. Significant variation of the physiological performance was not observed in the plants under control condition, because the values of the traits, except for SPAD, were not significantly different to each other. However, compared to control condition, all physiological performances were degraded in the plants under Cd-stress conditions, whereas significantly more degradation was observed in WT than in the transgenic plants (Figure 5A–C), as observed for plant growth traits. These data further support the increased tolerance of the latter to Cd stress than the former.

### 3.4. Ethylene Production and Expression Profiles of Its Biosynthesis and Receptor Genes

**Figure 6:**
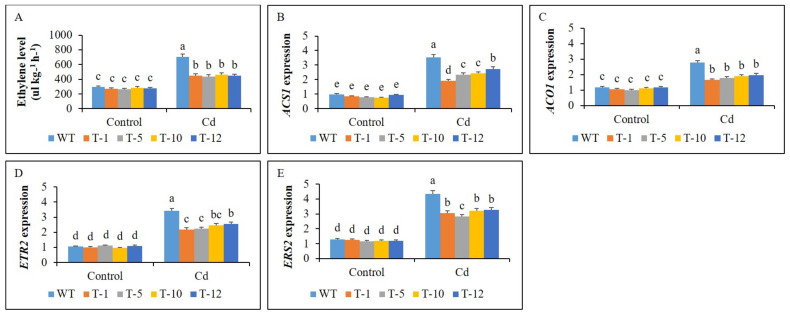
Comparison of ethylene production (**A**) and transcript levels of genes associated with ethylene biosynthesis and signaling (**B**–**E**) in the leaves of WT and transgenic petunia cv. ‘Mirage Rose’ plants under control and cadmium (Cd) stress conditions. Data were collected on the 30th day of the experiment. Data represent the means of three replicates and error bars indicate standard error. Means with the same letters are not significantly different by least significant difference test (LSDT, *p* < 0.05).

Ethylene production between WT and transgenic plants or within transgenic plants did not vary significantly under control conditions. When they were subjected to Cd stress, a significant elevation of ethylene levels was observed, whereas the elevation was significantly greater in the WT plants than in the transgenic plants (Figure 6A). This was further confirmed by detecting the expression levels of *ACS1* and *ACO1* in the leaves of the plants. *ACS1* and *ACO1* were not strongly expressed in the plants under control conditions, and their expression levels were not significantly different from each other. As expected, the expression levels were significantly elevated in the plants under Cd stress, and the expression levels detected in WT plants were significantly higher than that in the transgenic lines (Figure 6B,C). These results indicated that ethylene production was linked to the expression of its related genes in the plants under both conditions. As observed for *ACS1* and *ACO1*, the expression levels of *ETR2* and *ERS1* did not vary in the plants under control conditions. However, elevated expression of the genes was observed in all plants when exposed to Cd stress (Figure 6D,E), whereas their expression levels detected in WT plants were significantly higher than those in the transgenic plants.

### 3.5. Hydrogen Peroxide (H_2_O_2_) Accumulation in WT and Transgenic Plants

**Figure 7:**
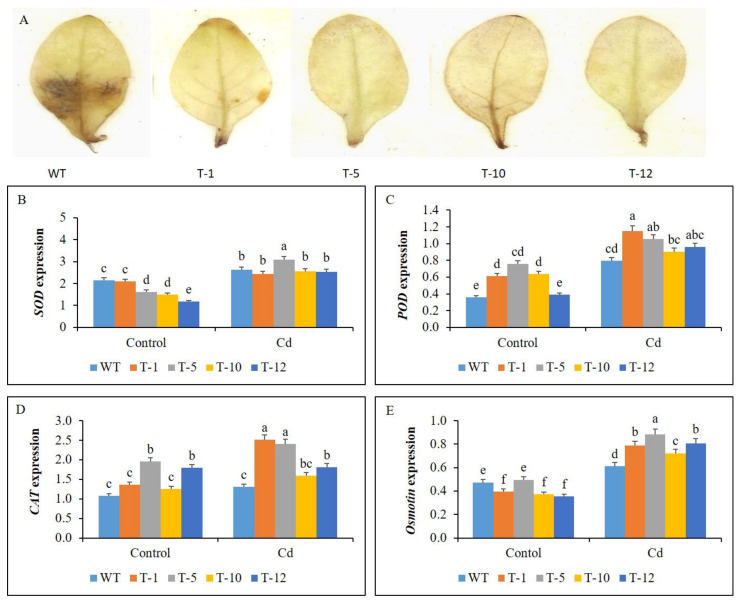
Illustration of hydrogen peroxide (H_2_O_2_) accumulation (**A**) in the leaves of WT and transgenic petunia cv. ‘Mirage Rose’ plants under cadmium (Cd) stress, and comparison of the expression levels of *SOD* (**B**), *POD* (**C**), *CAT* (**D**), and *Osmotin* (**E**) in their leaves under control and cadmium (Cd) stress. Data represent the means of three replicates and error bars indicate standard error. Means with the same letters are not significantly different by least significant difference test (LSDT, *p* < 0.05).

The accumulation of H_2_O_2_ in the leaves of the plants subjected to the stress for 30 days was detected using 3,3-diaminobenzidine (DAB) staining. No dark spots were observed in the leaves of the transgenic plants, but these were clearly seen in the leaves of the WT plants (Figure 7A), suggesting a higher accumulation of ROS in the leaves of WT plants.

### 3.6. Expression Profile of Antioxidant- and Proline-Related Genes

Under control conditions, differential expression of the antioxidant genes (*SOD*, *POD*, and *CAT*) and the proline-related gene (*Osmotin*) was observed in the plants. Specifically, the expression levels of *SOD* and *Osmotin* were higher in the WT than in most of the transgenic plants. However, the *CAT* and *POD* levels detected in WT plants were significantly lower than those in some transgenic plants. When the plants were exposed to Cd stress, their expression levels were significantly elevated compared to those in control conditions, except for WT and T-10 for CAT (Figure 7B–D). However, the elevation of the genes was significantly higher in the transgenic plants than in the WT plants, but there was no significant variation in *SOD* expression between the WT and transgenic plants, except for T-5.

### 3.7. Cd Concentration and Expression of GST and PCS

**Figure 8:**
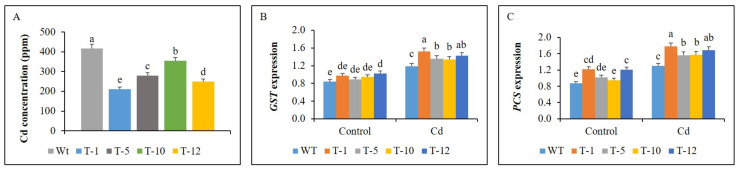
Comparison of Cd concentration (**A**) in WT and transgenic petunia cv. ‘Mirage Rose’ plants under cadmium (Cd) stress, and comparison of the expression levels of GST (**B**) and *PCS* (**C**) in their leaves under control and cadmium (Cd) stress conditions. Data represent the means of three replicates, and error bars indicate standard error. Means with the same letters are not significantly different by least significant difference test (LSDT, *p* < 0.05).

The concentration of Cd in the plants subjected to Cd stress for 30 days was evaluated. Results shown in Fig. 8A indicated that the content of Cd accumulated in WT plants was significantly higher than that in transgenic plants. In addition, slight or significant variations in Cd concentration within the transgenic lines were observed. The lowest Cd concentration was observed in T-1, followed by T-12 and T-5, and the highest concentration was observed in T-10 (Figure 8A).

When detecting the expression levels of the genes *GST* and *PCS,* which encode the metal chelation enzymes (glutathione S-transferase and phytochelatins), in the leaves of WT and transgenic plants, their expression levels were not significantly different to each other, except T-12 for GST and PCS and T-1 for PCS under control condition. Significant Cd-induced upregulation of the genes was observed in the plants exposed to Cd stress, whereas their expression was significantly higher in the transgenic plants than in the WT plants (Figure 8B,C).

## 4. Discussion

As petunias are highly sensitive to ethylene, their flower longevity is negatively affected by high ethylene production [1,2,3]. Overexpression of *acdS* significantly reduced ethylene production in tomato and canola [8,9,10,11], whereas *acdS* significantly extended shelf-life of tomato by reducing ethylene production [10]. Recently, we also observed that overexpression of *acdS* significantly reduced ethylene production in the leaves of the transgenic petunia cv. ‘Mirage Rose’ [4]. However, we did not investigate whether the *acdS* overexpression reduces ethylene production in floral tissues and improves flower longevity of the transgenic petunia. In addition, petunias, as bedding plants, are mostly grown in natural soil in open fields; in general, natural soils have a high possibility of being contaminated with HMs, including Cd, which induces ethylene production and inhibits plant growth. We did not assess the role of *acdS* in tolerance to Cd stress as well in our previous work. Therefore, in this study, we investigated whether the transgenic petunia cv. ‘Mirage Rose’ overexpressing *acdS* exhibited a greater flower longevity and improved tolerance to Cd stress.

In this study, *acdS* significantly lowered ethylene production in floral tissues at three different flower stages compared to those in WT. This could be attributed to the ability of *acdS* to break down the excess ethylene precursor ACC into ammonia and α-ketobutyrate in floral tissues [6,7]. We proved the ability of *acdS* to reduce the ACC levels by detecting the expression levels of ethylene-related genes in the floral tissues, whereas expression levels of ethylene biosynthesis genes (*ACS1* and *ACO1*) analyzed at all stages were significantly lower in the transgenic flowers than in the WT flowers. This validated the presence of low ACC levels in the former due to *acdS* expression because ACC level is positively linked to the expression of ethylene biosynthesis genes as well as ethylene production. This in turn leads to a decrease in expression levels of ethylene receptor genes (*ERS2* and *ETR2*), as the receptor genes were transcriptionally lower in the transgenic lines compared to WT. It indicated that ethylene production or expression of ethylene biosynthesis genes directly affected the expression of ethylene receptor genes. Reduction of ethylene production in the transgenic floral tissues led to improvement of flower longevity. An association between lower ethylene production and lower expression of ethylene biosynthesis genes or lower ethylene production and improved flower longevity has been previously reported in petunias [1,2,3]. Variation of ethylene production and flower longevity among the transgenic lines were also linked to the expression patterns of ethylene biosynthesis and receptor genes. Perhaps, the variation could be due to the differences in expression levels of *acdS* in the transgenic lines, as the expression levels can affect its ability to breakdown ethylene precursor ACC in the floral tissues, causing variation of ethylene biosynthesis genes at transcript levels and ethylene production. These findings highlight the possibility of using *acdS* to increase the flower longevity of petunias. Reduction of ethylene production and delaying of fruit ripening by *acdS* overexpression had been reported in canola and tomato [10,35]. However, *acdS* had not been overexpressed in ornamental plants, except the one done by Klee and Kishore [36], in which they also did not investigate the role of *acdS* in the improvement of flower longevity by assessing ethylene production and expression of ethylene-related genes in floral tissues. Therefore, this is the first report of revealing the ability of *acdS* to increase the flower longevity of the ornamental plant.

When the transgenic plants and WT plants were grown under control and Cd-stress conditions, the performance of plant growth and physiological traits was inferior in the stressed plants compared to those in the non-stressed plants (control), indicating the inhibitory effect of Cd on plant physiology and growth. Generally, high concentrations of Cd are toxic to plant tissues and induce excessive ethylene production, which in turn disrupts plant physiological functions through alterations in biochemical and molecular mechanisms [37,38]. The disruption of physiological functions reduces photosynthesis and the uptake of necessary nutrients, leading to the inhibition of plant growth [37,38]. Therefore, in this study, the impairment of plant physiological and growth performance in the Cd-stressed plants compared to the control could be attributed to the induction of high ethylene levels in Cd-stressed plant tissues. As expected, ethylene production and the expression levels of *ACS1* and *ACO1* were higher in the Cd-stressed plants than in the control, indicating that plant growth inhibition under Cd stress was because of Cd-induced ethylene production through the upregulation of ethylene biosynthesis genes. Schellingen et al. [24] claimed that the Cd-induced increase in ACC enzyme and ethylene biosynthesis in *Arabidopsis* was mainly due to upregulated *ACS2* and *ACS6* expression, as Cd neither induced ethylene nor inhibited plant growth in *Arabidopsis* when *ACS2* and *ACS6* were knocked out. Similarly, Iakimova et al. [21] revealed that Cd-induced cell death was linked to ethylene production because the addition of aminoethoxyvinylglycine (AVG) or silver thiosulfate (STS), which blocks ethylene biosynthesis or the ethylene receptor, markedly reduced Cd-induced cell death. Cd-induced ethylene production and plant growth inhibition through the upregulation of ethylene biosynthesis genes have been reported in many plant species [15,16,19,20,21,22,23,24,25]. Moreover, upregulated expression of ethylene receptor genes (*ETR2* and *ERS2*) was observed in plants grown under Cd stress, indicating their involvement in alleviating the deleterious effects of Cd-induced ethylene production on plant growth. The involvement of signaling-related genes in HM-stressed plants has also been reported previously [24,39].

Under Cd stress, more impairment of plant growth and physiological parameters was observed in WT than in the transgenic lines, revealing the greater tolerance of the latter to Cd stress than the former. This could be attributed to the overexpression of *acdS* in the latter because it can inhibit Cd-induced ethylene production in plant tissues by breaking down of excess ethylene precursor ACC levels [6,7]. This hypothesis was supported by our data, with lower ethylene production and expression levels of *ACS1* and *ACO1* in transgenic lines than in WT plants. This data suggested the existence of low ACC levels in transgenic lines because ACC levels are positively linked to ethylene production and expression of its related genes. The role of *acdS* in suppressing Cd-induced ethylene production and mitigating growth inhibition has been demonstrated in a few plant species by inoculating Cd-stressed plants with PGPB expressing *acdS* [26,27,28]. In addition, transgenic plants overexpressing *acdS* exhibit a reduction in HM-induced ethylene production and growth inhibition [8,9,11,29]. Under Cd stress, *ETR2* and *ERS2* expression was higher in WT than in the transgenic plants, suggesting that WT strongly triggered the genes to alleviate the deleterious effects of Cd-induced ethylene, as ethylene production levels in the former were higher than those in the latter, further supporting the involvement of ethylene signaling in Cd-stress tolerance. Schellingen et al. [24] also reported the involvement of ethylene signaling in Cd-induced ROS signaling in *Arabidopsis.*

HM-induced ROS production has been reported in many plant species [22,30,31,32,40]. We also observed Cd-induced ROS accumulation in all the plants. Greater suppression of plant growth in WT than in the transgenic plants was likely associated with the ROS levels because the level accumulated in the former was higher than that in the latter. ROS accumulation could be linked to ethylene production because ethylene production was higher in the WT than in the transgenic plants. Liu et al. (2008) observed that Cd induced ROS production in tomato plants, and ROS accumulation was linked to Cd-induced ethylene production. Moreover, Cd-stressed plants triggered higher levels of antioxidant genes (*SOD*, *CAT*, and *POD*) and proline-related genes (*Osmotin*) than did the non-stressed plants. This suggests that the stressed plants required more antioxidant and proline activities to scavenge Cd-induced excess ROS to sustain their growth against Cd stress. The involvement of proline and antioxidant enzyme activities or their related genes in HM stress tolerance has been previously reported [12,22,30,31,32,40]. One possible reason for the greater tolerance of the transgenic lines to Cd stress than the WT could be attributed to the presence of stronger antioxidant and proline activities in the former than in the latter, because expression of the antioxidant- and proline-related genes was higher in the former than in the latter.

In addition, greater growth inhibition in WT plants over the transgenic lines was linked to Cd concentration in the plants because its concentration in WT plants was significantly higher than those in the transgenic lines. The expression levels of *GST* and *PCS* involved in metal chelation were higher in transgenic plants than in WT plants, suggesting the presence of higher GST and PCS activities in the former than in the latter. Stronger triggering of the *GST* and *PCS* in transgenic plants compared to WT plants could be explained by the fact that the transgenic plants overexpressing *acdS* were less damaged by the effect of Cd-induced ethylene compared to WT plants; thus, they can trigger the genes stronger than those in the WT plants. Therefore, lower accumulation of Cd in the transgenic plants than in the WT plants and/or the greater tolerance of the transgenic plants to Cd stress than the WT plants could be attributed to the presence of higher GST and PCS activities in the former than in the latter. Ai et al. [12] also reported higher expression of *GST* and lower Cu accumulation in petunias. Moreover, overexpression of these genes also reduces HM accumulation and enhances tolerance to HM stress in *B. juncea*, *Arabidopsis*, *Populus canescens*, and *Nicotiana tabacum* [41,42,43,44]. Taken together, *acdS* overexpression in petunias enhanced flower longevity by reducing ethylene production and related gene expression in the floral tissues. Additionally, *acdS* overexpression significantly improved tolerance to Cd stress by reducing ethylene production in plant tissues via downregulation of ethylene-related genes, Cd accumulation via upregulation of metal chelation-related genes, and ROS accumulation via upregulation of antioxidant- and proline-related genes.

## 5. Conclusions

Transgenic petunias overexpressing *acdS* exhibited greater flower longevity and tolerance to Cd stress than WT plants. This was because of the role of *acdS* in reducing ethylene production in vegetative and floral tissues. Under Cd stress, the stressed plants triggered antioxidant- and proline-related genes to scavenge the ethylene-induced ROS in the plant tissues, whereas the higher gene expression in the transgenic plants than in the WT revealed a plausible reason why the former has a greater tolerance to Cd stress than the latter. Moreover, the lowering of Cd accumulation in the transgenic plants compared to WT plants was due to the role of *acdS* in stronger triggering of metal chelation genes in the former than in the latter. Our findings suggest a mechanism by which *acdS* improves tolerance to Cd stress. We expect that *acdS* overexpression in ornamental bedding plants would extend flower longevity by reducing ethylene production in floral tissues and alleviating the effect of HM-induced ethylene on growth inhibition when they were grown in HM-contaminated soil.

## Data Availability

Not applicable.

## Supplementary Materials

The following are available online at https://www.mdpi.com/article/10.3390/cells11203197/s1, Table S1: Primers used for gene expression analysis using quantitative real-time PCR.

## Abbreviations

WT: wild-type; HMs, heavy metals; *ACS*, 1-aminocyclopropane-1-carboxylic acid synthase; *ACO,* 1-aminocyclopropane-1-carboxylic acid oxidase; PGPB, plant-growth promoting bacteria; *ACCD*, 1-aminocyclopropane-1-carboxylic acid deaminase; ACC, 1-aminocyclopropane-1-carboxylic acid; ROS, reactive oxygen species; MS, Murashige and Skoog; qPCR, real-time polymerase chain reaction; *ETR2*, ethylene resistant 2; *ERS2,* ethylene response sensor 2, SOD, superoxide dismutase; CAT, catalase, GST, glutathione S-transferase; PCS, phytochelatin synthase; POD, peroxidase.
