# Supplementary material for: Overexpression of acdS in Petunia hybrida Improved Flower Longevity and Cadmium-Stress Tolerance by Reducing Ethylene Production in Floral and Vegetative Tissues
Source: Cells. 2022 Oct 11;11(20):3197. doi: 10.3390/cells11203197 (PMC9600315; doi:10.3390/cells11203197)
Supplement: Supplementary file 1 [file cells-11-03197-s001.zip › cells-1946706-supplementary.pdf]

Supplementary Material

Supplementary Table S1. Primers used for gene expression analysis using quantitative real-time PCR.

Gene	Accession No.	Primers sequences	
		Forward primer **(5' to 3')**	Reverse primer **(5' to 3')**
ACS1	Z18953.1	5'-ACCGAGTAGTTATGGCCGGT-3'	5'-GAATGCATCGCCAGCATCAG-3'
ACO1	L21976.2	5'- ATCAGCTTGGACAAAGTGAATGG-3'	5'-CACCAACTCAAAGAAGCCCC-3'
ETR2	DQ154119.1	5'-GAGATAGTCAGGGTCGTGGC-3'	5'-AGCTCGATTTTGCTCCTCCA-3'
ERS2	DQ154118.1	5'-TGGGTCCTCATGCAGTTTGC-3'	5'-CATAACCACAGCGACCGTCT-3'
SOD	X14352.1	5'- ACTGCTCCGTCACCCAAAAC-3'	5'- TGGTAAGGCTGAGTTCGTGG-3'
POD	D11396.1	5'- ACTGCTCCGTCACCCAAAAC-3'	5'- GCCCTGGTTGCTTAAGTC-3'
CAT	AY726007.1	5'- CAGCCAGTGGGACGATTAGT-3'	5'- GGCACCACAATAGAAGGGCA-3'
Osmotin	AF376058.1	5'- CTTTCGCCCCAACTAAGCCT-3'	5'- TGCACCAGGACATTCACCAT-3'
GST	NM_001325692	5'- CGCAAAGGAGAGGAGCAAGA-3'	5'- TGTCACCCGCAAAGAATTTCT-3'
PCS	KP136425.1	5'- GGGCAATTCTGCTACAGTGG-3'	5'- GACCAGAACATCAACCCCCT-3'
Tubulin	SGN-U207876	5'-TGGAAACTCAACCTCCATCCA-3'	5'-TTTCGTCCATTCCTTCACCTG-3'

PCR condition - 95℃(10min) followed by 40 cycles of [95℃(15s)-59℃(1min)] -95℃(15s)-60℃(1min)-95℃(15s)
